# Biological strategy for the fabrication of highly ordered aragonite helices: the microstructure of the cavolinioidean gastropods

**DOI:** 10.1038/srep25989

**Published:** 2016-05-16

**Authors:** Antonio G. Checa, Elena Macías-Sánchez, Joaquín Ramírez-Rico

**Affiliations:** 1 Departamento de Estratigrafía y Paleontología, Universidad de Granada, Granada, 18071, Spain; 2 Instituto Andaluz de Ciencias de la Tierra, CSIC-Universidad de Granada, Armilla, 18100, Spain; 3 Departamento de Física de la Materia Condensada, Universidad de Sevilla, 41012, Sevilla, Spain; 4 Instituto de Ciencia de Materiales de Sevilla (CSIC-Universidad de Sevilla), 41092 Sevilla, Spain

## Abstract

The Cavolinioidea are planktonic gastropods which construct their shells with the so-called aragonitic helical fibrous microstructure, consisting of a highly ordered arrangement of helically coiled interlocking continuous crystalline aragonite fibres. Our study reveals that, despite the high and continuous degree of interlocking between fibres, every fibre has a differentiated organic-rich thin external band, which is never invaded by neighbouring fibres. In this way, fibres avoid extinction. These intra-fibre organic-rich bands appear on the growth surface of the shell as minuscule elevations, which have to be secreted differentially by the outer mantle cells. We propose that, as the shell thickens during mineralization, fibre secretion proceeds by a mechanism of contact recognition and displacement of the tips along circular trajectories by the cells of the outer mantle surface. Given the sizes of the tips, this mechanism has to operate at the subcellular level. Accordingly, the fabrication of the helical microstructure is under strict biological control. This mechanism of fibre-by-fibre fabrication by the mantle cells is unlike that any other shell microstructure.

Molluscs constitute a major calcifying group, second only after the arthropods within the marine realm. They construct their shells with a series of microstructures, which display outstanding biomechanical properties, such as toughness (nacre, crossed lamellar), elasticity (columnar prismatic), light-weight (chalk), or softness plus fracture localization (foliated calcite plus chalk). On this basis, they can be qualified as functional materials. These properties result from the combination and interaction between the two main constituents (calcium carbonate crystals, organic matter), which are otherwise relatively weak. A key goal is to decipher the biological strategies leading to such sophisticated biocomposites, as a means, among other things, for material scientists to use them as inspiration for advanced functional materials.

The most profusely studied and well-known microstructures include nacre, prismatic (either calcitic or aragonitic), crossed-lamellar and foliated, because of their mechanical properties and widespread distribution within the group, although the molluscs produce more than ten main varieties. In all cases, some relationships between the microstructures and their equivalent inorganic counterparts can be established on the basis of e.g. crystalline shapes (prisms, fibres, laths, etc.). There is nevertheless one microstructure that stretches these boundaries and looks like anything but a crystalline aggregate. This is the so-called aragonitic helical fibrous microstructure (AHFM)^1^,^2^, made up of very thin aragonite fibres which coil helically for several turns along an axis perpendicular to the shell’s surface (Fig. 1a–f). This microstructure has a high degree of internal order in the sense that all helices display similar shapes and orientations. Despite its intricacy, it is a minor microstructure in the sense that it has been reported only in species of the superfamily Cavolinioidea^3^. This is a reduced group of small planktonic gastropods, with four living families, eight genera and 52 species^4^. In relation to their planktonic habits, the shells are very thin and transparent, and their morphologies depart markedly from the coiled shells of most gastropods, being straight cone-, vase- or more oddly shaped (Supplementary Fig. S1). The shells of all cavoliniodeans are made up of AHFM^5^, although in some, crossed lamellar layers (composed of straight fibres crossing at an angle) can also be found in an external position^6^. This group is a sister taxon of the regularly coiled Limacinoidea (the shells of which are made up of the crossed-lamellar microstructure) (Supplementary Fig. S1), and both comprise the monophyletic suborder Euthecosomata^7^. The shells of the Euthecosomata are particularly sensitive to rising levels of dissolved CO_2_, and it has thus become a target group of studies aiming to evaluate the future impact of rising CO_2_ atmospheric levels on calcifying organisms^8^,^9^,^10^.

Although the AHFM was first described more than 40 years ago^1^, it was not until the present decade that it has re-attracted the attention of materials scientists because of its unusual fabric, crystallography, and biomechanical performance. It has been shown that cracks follow tortuous paths around the contours of the helical fibres, thus effectively contributing to energy dissipation^11^. Previous crystallographic studies^6^,^11^,^12^ have shown that the *c*-axes of the aragonite crystals are strictly aligned with the coiling axes of the evenly aligned helical fibres. Recently, it has been shown that the crystals in *Cuvierina* are also relatively well aligned in the *a*-*b* plane and that they tend to grow preferentially along the projection of the *b*-axis on the fibre axis^13^. When this preferential crystallographic growth direction substantially departs from the actual growth direction of the fibre due to the permanent curling, abrupt changes in the orientation of the *a*- and *b*-axes across {110} twins permit growth to continue in the desired direction^13^. This study evidences that this is the mechanism by which fibres adapt their crystallography to helical growth and not vice-versa, i.e. it does not guide helical growth. Hence, we must assume that the helical growth has to be achieved by some form of unknown growth strategy.

In this study, we revisit the morphological features of the AHFM by means of high-resolution techniques. Some features are completely unlike those of other molluscan microstructures and provide evidence that the organism strictly dictates the path of every fibre through an intricate and elaborate strategy, thereby exerting a degree of control not found in any other microstructure. This is the first time that such a subtle organic control mechanism for the fabrication of a microstructure has been brought to light.

## Results

### Morphology of fibres

**Figure 1:**
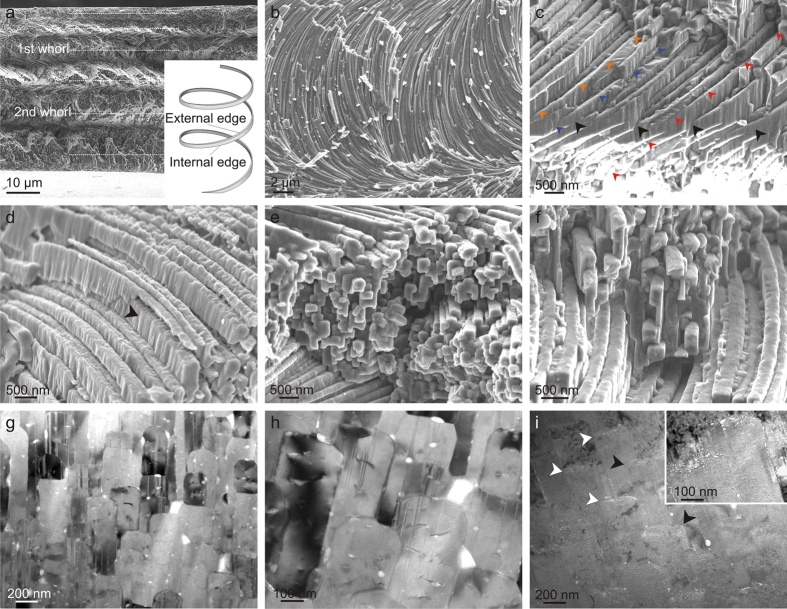
Fracture (SEM) and section (TEM) views of the AHFM of the Cavolinioidea. (**a)** Radial section of the shell of *Cuvierina columnella*. Horizontal broken lines are spaced at about half a turn. In this specimen, the helices complete some three turns. Note how the spacing between half turns (and, thus, the lead angle) increases towards the shell interior. The inset is a sketch of the ideal morphology of one such helical fibres. (**b)** Top view of a fracture through the shell of *Cuvierina columnella*, approximately parallel to the shell surface. The observed changes in orientation of fibres are caused by the unevenness of the fracture surface. (**c**) Fracture perpendicular to the surface of the shell of *Cuvierina columnella*, showing the interpenetration between fibres (black arrows). Colour arrows show the paths of three individual fibres (in different colours). (**d**) Etched fracture through the shell of *Cavolinia longirostris*. Etching permits a view of the complete process of crossing between two fibres; the lower fibre (arrow) crosses the upper fibre completely from right to left. (**e,f**) Transversely fractured fibres of *Clio pyramidata* (**e**) and *Cuvierina urceolaris* (**f**), showing the complex outlines caused by interlocking. (**g**) Transversal section of the fibres of *Cuvierina columnella*, exposing their interlocking cross-sections. The vertical thin lines within the fibres are twinning planes. (**h)** Detail of an area similar to (**g)**. (**i**) Transversal section of the fibres of *Cuvierina columnella*. Fibres consistently show an external band (~150–200 nm thick) with an unusual density of white spots. The inset is a close-up of two such fibres. Arrows point to some examples. The external shell surface is to the top in (**a**) and (**c)** to (**i**).

Little can be added to the detailed descriptions of Bé *et al.*^1^ and subsequent authors^2^,^6^,^11^,^12^,^13^ about the general morphology of the fibres. In all the species examined, the fibres invariably coil dextrally. They complete a variable number of turns, depending, though not strictly, on shell thickness, from less than one turn in *Creseis clava* (shell thickness ~12 μm) to up to more than three turns in *Cuvierina columnella* (shell thickness ~52 μm) (Fig. 1a, Supplementary Fig. S2). The axes of the helices are invariably perpendicular to the shell surface and the orientation of fibres is approximately (though not strictly) the same at a particular depth within the shell (Fig. 1a, Supplementary Fig. S2)^1^,^12^,^13^. When the fibres can be seen curving in fractures parallel to the shell surface (Fig. 1b), it is because the fracture has in fact partly followed the path of the helices.

In general, the helix angle is noticeably smaller for the first turn (12–15°) and later increases (to 25°–30°) towards the shell interior^1^,^13^ (Fig. 1a, Supplementary Figs S2 and S7). In *Creseis*, the small number of whorls does not permit similar observations (Supplementary Fig. S2). The helix radius can be roughly estimated at between 12 and 16 μm and we have not been able to confirm differences among the various species; in single specimens, this radius remains constant throughout the shell thickness. SEM observations show that fibres are continuous all along their exposed lengths (Fig. 1b–f), i.e. up to haf a turn (Fig. 1c, Supplementary Fig. S3).

**Figure 2:**
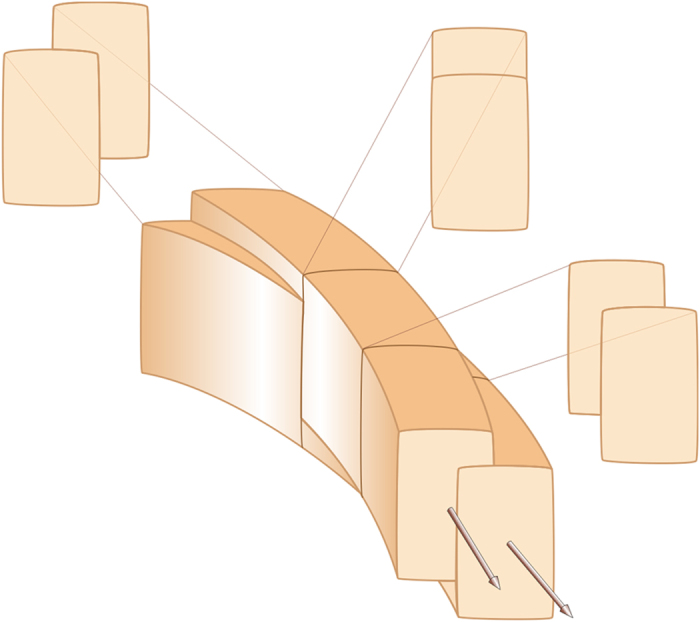
Interlocking mechanism between fibres. Two non-coaxial spiral fibres interlock progressively in the way shown above. Both fibres grow towards the reader and towards the bottom (arrows). The lower fibre crosses the upper fibre (the one running ahead) from left to right. During the process, the cross-sectional outline of the upper fibre changes progressively in the way depicted.

One of the most unusual aspects concerns the interlocking of fibres. This occurs because fibres at a distance of less than two-fold the helix radius intersect twice per turn, which is particularly evident in vertical fracture (Fig. 1c). After crossing each other, fibres reappear and continue to grow (Fig. 1d). This process accounts for the complexity of the angular cross-sectional outlines of fibres^1^,^11^,^12^, which is evident both in fractures under the SEM (Fig. 1e,f) and in TEM sections (Fig. 1g,h) of the fibres at high angles to their long axis. The process of interlocking causes unavoidable permanent changes in the outlines of the fibres (Fig. 2). It is particularly striking that the shapes of the cross-sectional outlines are not random. In particular, the external edge (from here on, we will call ‘external’ edge or band that pointing towards the external shell surface; Fig. 1a, inset) in all cases remains unaffected, and it is only below a certain distance from the external edge (roughly estimated at between 100 and 200 nm) that a given fibre is interlocked from either one or the two sides (Fig. 1e–h).

Diffraction contrast images illustrate the dense arrangement of {110} twins, shown in previous studies^11^,^13^, with incoherent twinning boundaries being frequent (Fig. 1g,h, Supplementary Fig. S4). Twin planes extend all along the fibre height in parallel to the helix axis (Fig. 1g,h, Supplementary Fig. S4). In some instances, fibre splitting is evident under the TEM^13^. Some cases in which twin boundaries revert to crystal boundaries have also been recorded (Supplementary Fig. S4). In one *Cuvierina columnella* specimen the fibres showed an external band 100–200 nm thick full of bright spots (Fig. 1i); an identical feature has previously been reported in *Clio pyramidata*^11^.

### Internal shell-growth surface

**Figure 3:**
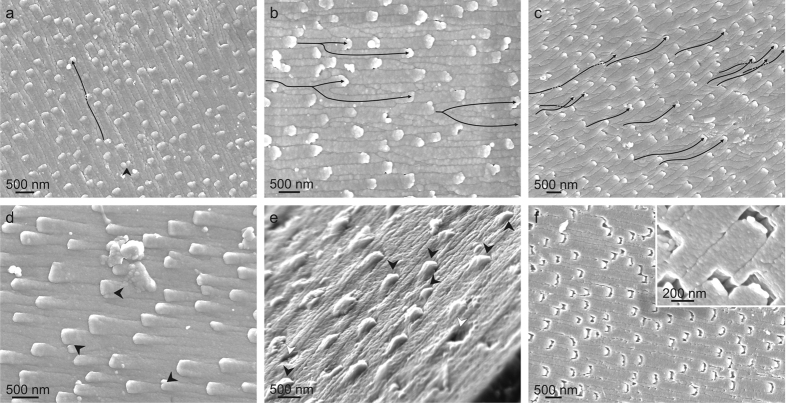
Features on the internal shell (growth) surface of the Cavolinioidea as revealed by SEM. (**a–c)** Slightly bleached specimens of *Cuvierina columnella* (**a**,**b**), and *Cuvierina urceolaris* (**c**), showing frequent examples of changes in orientation of the fibres (some trajectories are indicated with arrows in (**a**,**c**)), fibre interception and reappearance (partly broken arrows in (**a**,**c)**), and fibre division (coarse arrow in (**a)** and splitting arrows in (**b**)). (**d**) *Cavolinia inflexa*. Several tips are in the process of division (arrows). Note also the linear or angular anterior outline of the tips. (**e**) Oblique close-up view of *Cuvierina columnella*. Some tips (black arrows) have a planar adoral slope (black arrows). White arrows point to missing tips. (**f**) Heavily bleached shell of *Cuvierina columnella*. The tips of all fibres have dissolved preferentially. The close-up view (inset) shows how the dissolution has penetrated inwards towards the shell interior.

In all species examined, the outlines of the individual fibres on the growth surface are easily discernible (Fig. 3). The outlines of fibres on the growth surface are notably elongated in parallel to the growth direction of the fibres (Fig. 3a–d). In general, fibres appear evenly aligned (Fig. 3a,b,d), although there are instances in which fibres show some degree of twisting and the alignment is much poorer (Fig. 3c). Some fibres cut completely across the path of others, but this is only in appearance, since the affected fibres reappear later in the growth direction. The frequency of pseudo-extinction events increases with the degree of misalignment (compare Fig. 3a to c). Occasionally, fibre-splitting events are recorded (Fig. 3a,b,d). In line with the observations made on fractures either parallel or perpendicular to the shell surfaces (Fig. 1b–f), no unequivocal case was found in which a fibre either stops growing or is initiated *de novo*.

**Figure 4:**
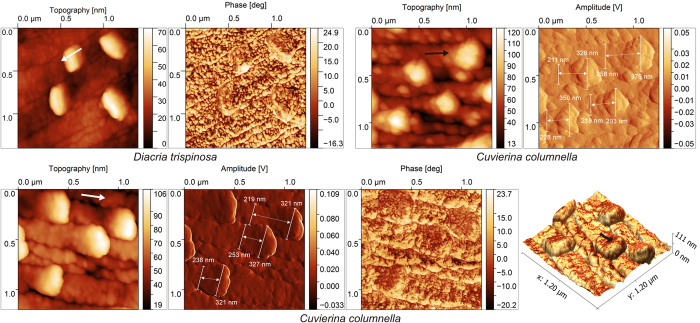
AFM views of the internal growth surfaces of the species indicated. The phase and amplitude views show that the surface texture of the fibres is composed of nanogranules, which are separated by high-contrast interspaces. The texture changes at the tips of the fibres, where granules are coarser and high-contrast interspaces fade out. The marked increase in width from the fibre main bodies to the tips is demonstrated by the measurements provided in the two amplitude views of *Cuvierina columnella* (top right and bottom central-left panels). In coincidence with the topography images, the 3D reconstruction (bottom right panel) shows how the fibres elevate progressively at the position of the tips. Arrows indicate the growth direction of the fibres. In the 3D reconstruction height is proportional to topography data while the overlay texture comes from the phase data.

A distinctive character is the presence under the SEM of minute bulges at the very anterior end (tip) of the fibres (Fig. 3a–e). They have been recognized in all species examined, although the aspect may change slightly (compare Fig. 3a to d). They appear as tiny elevations with a high, differentiated anterior (in relation to the growth of fibres) slope and much lower posterior slope. In some cases, the anterior outline is linear or pointed (Fig. 3d) and the slope is planar (Fig. 3e). They also bleach differentially, leaving an empty band of ~100 nm in width, which penetrates towards the shell interior along the external part of the fibre (Fig. 3f). AFM observations offered additional insight. The posterior slope elevates progressively from the fibre surface, whereas the anterior slope is more or less constant and markedly higher (Fig. 4, Supplementary Fig. S5). Longitudinal profiles of the anterior ends of the fibres provide evidence that the tips rise above the shell growth surface by 30 nm in *Clio pyramidata*, 50–60 nm in *Diacria trispinosa* and 65–85 nm in *Cuvierin*a columnella (Supplementary Fig. S5). Tips easily detach from the rest of the fibre, usually leaving a flat, possibly crystalline, surface (Supplementary Fig. S5). As is typical in biocrystals^14^, the surface texture of the fibres is nanogranular^13^, with nanogranules being separated by high-contrast lines or patches (Fig. 4). However, tips are composed of nanogranules that are larger (up to 200 nm wide) than those of the rest of the fibre surface (30–100 nm wide), providing the former with a smoother aspect (Fig. 4).

In well-preserved specimens, the width of the fibres increases markedly (by 30–60%) towards the position of the tip, in coincidence with the elevation (Fig. 4). In this way, laterally, they slightly overlap the adjacent fibres. Oriented TEM sections show that there is no apparent overlap in the anterior direction, and the tips touch the shell surface at the point of emergence of the fibre in front (Supplementary Fig. S6).

### Decalcified samples

**Figure 5:**
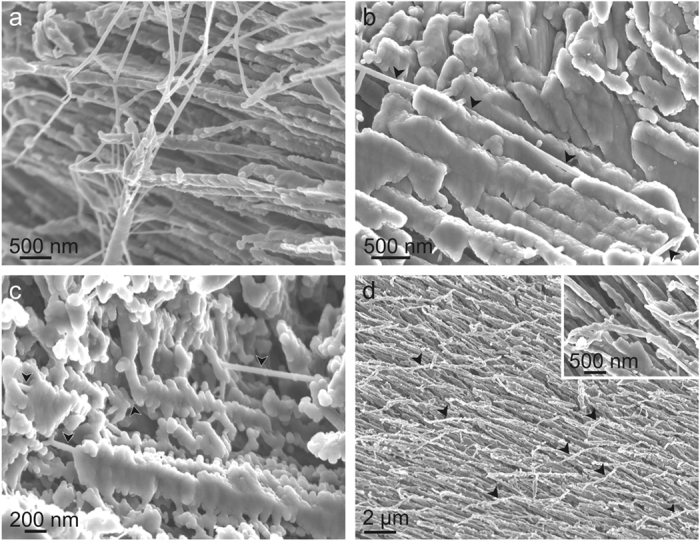
SEM views of semidecalcified fractures of the shells of the Cavolinioidea. (**a**) *Cavolinia longirostris*, showing the network of organic threads left after decalcification. (**b**) *Cuvierina columnella*. Long organic thread (>20 μm long, total length not shown) crossing a semidecalcified fibre through its external part (arrows). (**c**) *Cuvierina columnella*. Arrows point to organic fibres that have remained more or less *in situ*; they consistently enter the corresponding fibres close to their external surfaces. (**d**) Specimen of *Cavolinia longirostris* in which the dissolution has not completely exposed the organic fibres, which are still surrounded by calcium carbonate granules. They appear as flexible thin extensions of the top parts of the mineral fibres, which have deflected leftwards during the treatment (some cases are indicated with arrows). The inset is a detail. (**a**,**d**) are views of the surfaces of the fibres looking towards the external shell surface. (**b**,**c**) are lateral views with the external shell surface to the top.

Semi-decalcified samples show the presence of relatively abundant organic remains, in the form of fully organic threads (40–60 nm wide and up to tens of μm long) (Fig. 5a). When their original position has not been fully altered (e.g. by adhering to other fibres), it can be seen that the organic threads consistently emerge from and align with the top part of the fibres (Fig. 5b,c). In some cases in which the fibres were not complete denuded, they occur as alignments of calcium carbonate globules in continuation of the fibres, revealing a highly flexible behaviour (Fig. 5d).

## Discussion

The AHFM microstructure of cavolinioideans is unique in being composed of helical fibres that run from the external to the internal shell surface at the same time as they coil helically. The growth surfaces of the fibres on the inner shell surface show a high degree of alignment. This implies that in these gastropods the calcification of the shell proceeds from the external to the internal shell surface, where the mantle adheres^6^. This is clearly unlike the situation in trochospiral gastropods in which calcification proceeds mainly in the adoral growth direction.

Although we cannot provide conclusive evidence that fibres are fully continuous throughout the shell thickness, no cases of interruption have been noted in our or other published material^1^,^6^,^12^,^13^ along the exposed lengths of the fibres in fractures parallel to the helix axes (Fig. 1a–f, Supplementary Fig. S3). In line with previous authors^1^,^12^,^13^, we assume full continuity of the fibres.

**Figure 6:**
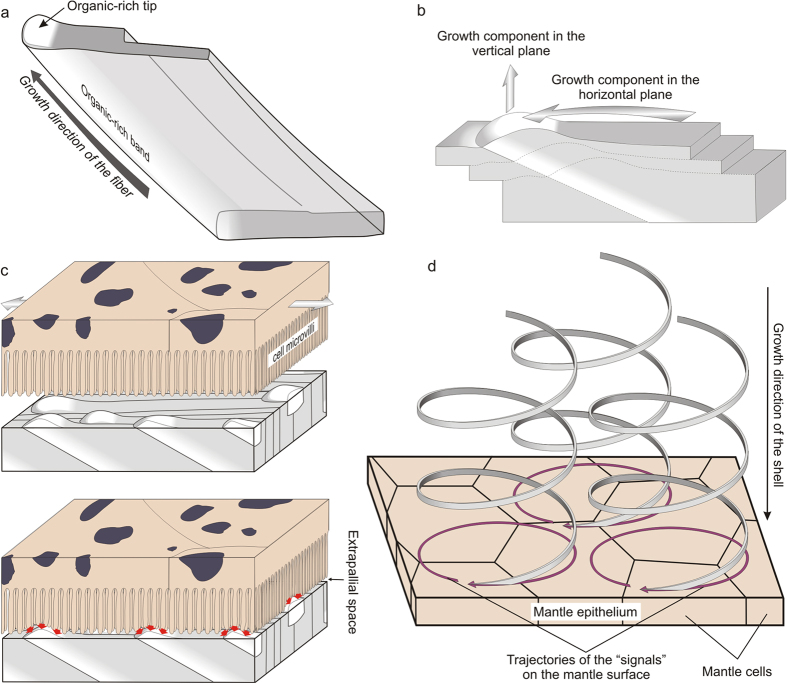
Proposed mechanism of fabrication of the AHFM. (**a**) Model for the ultrastructure of a fibre. The fibre segment is seen obliquely from its growth surface. With fibre growth, the elevated organic-rich tip generates an anterior growth band, whereas the rest of the fibre volume is homogeneous in composition. (**b**) Mechanism of movement of a fibre in three different time planes. From one plane to the next, the fibres move in the direction of the tips at the same time as they rotate slightly to the left (due to the dextral helical coiling). Since the tip emerges from the background surface at any time, it can never be overgrown by the surrounding fibres. (**c**) The contact recognition mechanism. During non-secretory periods (upper sketch), the mantle is able to move with respect to the shell in an anterior-posterior direction (arrows). The shell-mantle separation is hypothetical. During shell secretion (lower sketch) the outer mantle epithelium adheres to the shell surface. Cell sensorial extensions are able to recognize the positions of the fibre tips (red arrows). (**d**) Model for the fabrication of helical fibres. During growth of the helices, the signal for the production of each tip follows a circular trajectory on the mantle surface. With the relative sizes of fibres and mantle cells taken into account, each trajectory moves both intracellularly and across neighbouring cells. (**a**–**c)** are oblique views from the internal shell surface, and (**d**) is an oblique view from the external shell surface.

Our observations as well as previous ones^1^,^6^,^11^,^12^,^13^ reveal that fibres permanently interlock, causing changes in their cross-sectional outlines. Nevertheless, instead of extinguishing due to repeated collision, the fibres survive and continue to grow. Understanding how fibres avoid competition is essential in order to accurately interpret the growth dynamics of fibres, a challenging question for material scientists. An important clue in this sense comes from the fact that, throughout this process, the full dimensions of the external strip (100–200 nm thick) of every fibre are maintained, i.e. no fibres were found to be worn away by neighbouring ones. Our treatment protocols reveal that this external band dissolves preferentially upon bleaching. In addition, partial dissolution by etching reveals the presence of longitudinal mucous strings, which emerge from this area, and that are presumably the result of agglutination of intra-crystalline organic matter. In TEM sections, this area sometimes contains a high density of white inclusions, which have been interpreted elsewhere as occluded biomacromolecules in other biological aragonites^15^,^16^ and calcites^17^,^18^,^19^. All this evidence together implies that the external bands are richer in organic matter than the rest of the fibres, although we cannot currently provide any quantification. The internal structure of a fibre is sketched in Fig. 6a. As explained above, the nanogranular texture of this area is coarser (Fig. 4), perhaps being a result of the higher organic content. In TEM sections of the shell interior, the frequent {110} twins extend all along the fibre thickness, including this area (Fig. 1g,h, Supplementary Fig. S4), implying that the whole fibre volume is crystalline.

On the growth surface, this organic-rich band emerges as a small bulge (tip), which is invariably placed in an anteriormost position (Figs 3 and 4). Present models of biomineralization imply that crystal growth proceeds through a transient ACC phase, which transforms in either aragonite or calcite by secondary nucleation^20^. There is thus the possibility that the tips emerge after post-mortem dissolution of an ACC cortex carpeting the internal shell (growth) surface. However, this is highly unlikely since, in order for the tips to have been initially masked, the required ACC cortex thickness should have been on the order of many tens of nm (see AFM heights in Supplementary Fig. S5). In the few reports on ACC in biogenic aragonite (nacre)^21^,^22^, the thicknesses are on the order of a few (<10) nm. Therefore, we infer that tips were present during the *in vivo* shell mineralization process.

The fact that the organic-rich band that emerges at the tip is never invaded by neighbouring fibres suggests that the formation of tips may be the means by which fibres somehow avoid extinction by competition. Except for the protruding tips, the growth surface of the shells of Cavolinioidea are even, with the only relief being the nanogranules forming the crystals (with elevations <15 nm; Supplementary Fig. S5). During calcification, the whole growth surface, including the elevations, moves inwards towards the shell interior (Fig. 6b). Note that, with fibre growth, the elevated tips also move anteriorly and laterally, thus following circular trajectories when projected on the growth surface (Fig. 6b). In this way, the tips become permanent elevations and cannot be displaced or overgrown by the neighbouring fibres. In this simple way, fibres avoid competition. As described above, the tips, as observed on the growth surface, are wider (Fig. 4) than after the corresponding area of the fibre has become incorporated within the shell with further growth (Fig. 3f). The reduction in volume would make sense if the tip initially consisted of a transient hydrated phase, particularly hydrated amorphous calcium carbonate (ACC), which subsequently transformed into aragonite (may be through an intermediate non-hydrated ACC phase). The size reduction detected fits the difference in molar volume between hydrated ACC [e.g. 54.1 cm^3^.mol^−1^ for CaCO_3_.H_2_O]^23^ and aragonite [34.13 cm^3^.mol^−1^]^24^. We have found no additional evidence in support of this hypothesis.

As explained above, fibre-splitting processes are not uncommon (Fig. 3a,b,d). On the contrary, no case was found in which fibres were outcompeted by others. This implies that the number of fibres present at particular growth planes (parallel to the shell growth surface) increases with time (i.e. with shell thickness). This may account for the observed increase in the lead angle from the exterior to the interior of the shell (Fig. 1a, Supplementary Figs S2 and S7), i.e. a higher lead angle implies that the fibres reach the growth surface at a higher angle; accordingly, their sections on this surface will have a smaller surface area (Supplementary Fig. S7). Therefore, a higher number of fibres can be accommodated within the same area of the growth surface towards more internal shell areas.

In microscopic view, the growth surfaces of molluscan shells are characterized by the presence of crystals or units forming the different microstructures (e.g. nacre, foliated, etc.; Supplementary Fig. S8). In no case are there differentiated subcrystalline structures, such as the emerging fibre tips described in Cavolinioidea, which constitute a unique feature among the molluscan microstructures. The only way by which differentiated tips could form is by direct secretion by the mantle. In particular, small regions able to secrete them must exist in the mantle. Tips measure 300–400 nm × 150–250 nm (e.g. Fig. 4); that is, they are much smaller than the diameter of a mantle cell [7–8 μm wide in ref. 6, Fig. 25]. This means that tips are secreted at a subcellular level. In molluscs, during the shell-formation process, the contact relationship between the mantle and the shell is not permanent. First, the mantle is able to move forwards and backwards with respect to the shell (Fig. 6c, upper scheme). In addition, cell death and division unavoidably changes the positions of cells over time. This implies that cells must be able to recognize or sense the position of the protruding tips in order to extend the pattern. Therefore, a contact-recognition mechanism must operate (Fig. 6c, lower scheme). What remains unclear is why the tips are enriched in organic molecules, unless this is part of the recognition system.

Since the tips move with time to generate (3D) helical fibres, the mantle cells must be able to propagate their secretion onto the 2D mantle surface. The movement required on this surface to generate a helix with a coiling axis perpendicular to the shell surface is along a circle with a radius equivalent to that of the fibre helix (Fig. 6d). The propagation of the signal necessary for the secretion of fibre tips along circular trajectories could proceed by reaction-diffusion processes of morphogens, which are widely known in cellular biology [see review in^25^]. In the AFHM case, it is striking that, given the tiny sizes of the tips, the signals have to travel within single cells and across the cell boundaries, without any apparent disturbance. This is unlike usual models for pattern formation based on reaction-diffusion mechanisms in which the signals travel across, but not within, cells. In summary, the contact recognition mechanism as well as the signal-transmission process must operate at a subcellular level.

Our cellular model is a plausible explanation in view of the existing evidence, but there are alternative explanations. Helical growth is common in nacre^26^, but the origin is through mineralization of a previous organic template^27^. Helical arrangements of nanocrystals of BaCO_3_ synthesised in the presence of block copolymers^28^, differ from the cavolinioidean fibres in that these are continuous, though twinned, crystals (Supplementary Fig. S4), with a continuous and well-defined growth front. In fact, Willinger *et al.*^13^ concluded that the helical growth pattern of cavolinioidean fibres is not dictated by crystal growth. Another likely possibility is that fibre growth proceeds through progressive calcification of a previous helical organic scaffold, organized according to a chiral nematic liquid crystalline phase, of the kind proposed for many biological patterns^29^, including molluscs^27^,^30^. In this view, the physically-driven orientation of (not observed) organic nanorods might provide the signal for the mantle cells to propagate the pattern, thus substituting for the above invoked activity of morphogens. Even if this were the case, fibres should be secreted individually by the mantle cells and their tips should continue to provide positional information.

## Conclusions

The secretion of the AHFM is driven by sophisticated mechanisms that operate at the subcellular level. Accordingly, this is under very strict biological control. Shell microstructures are aggregates produced in a constrained environment, i.e. the thin liquid film between the mantle and the forming shell (Fig. 6c), called the extrapallial space^27^,^31^,^32^,^33^. Their formation is governed by crystal growth processes, modulated by organic matrices. The AHFM is an exception because the mantle cells directly determine the growth trajectories of crystalline fibres.

Despite being a highly functional material^11^,^12^, the AHFM appeared only once in the history of molluscs [some 53 Ma ago, e.g.^3^] and in a much reduced group of gastropods. This evolutionary restriction might have to do with the complexity of its fabrication process, which necessarily involves a large genetic pool. On the contrary, nacre and the columnar prismatic layers of many molluscs, the formation of which largely involves physical processes of self-organization^27^, are widely used as structural materials for toughness and flexibility, respectively, and have appeared repeatedly in molluscs^34^,^35^ early in the history of the group. The difference in the evolutionary success between the two materials may lie in the required genetic complexity of the AHFM *vs.* the simplicity of nacre and prismatic layers. Nothing to date is known on how the most common material in molluscs, the crossed-lamellar, is constructed.

One of the aims of biomimetics is to reproduce biomaterials in the laboratory by taking natural processes as inspiration. The limitations are that purely biological processes (i.e. cellular activity) are not within the reach of current nanotechnology. Therefore, the probability of mimicking microstructures in the laboratory depends on how much their production is regulated by physical *vs.* biological processes. For instance, the future possibility of mimicking nacre [which is based largely on liquid crystallization^27^] will be much higher than that of the AHFM.

## Methods

### Material

Empty shells of *Diacria quadridentata*, *D. trispinosa, Diacavolinia longirostris, Cavolinia inflexa* (Family Cavoliniidae), *Clio pyramidata* (Cliidae), *Creseis clava* (Creseidae), and *Cuvierina columnella* (Cuvierinidae), all from the Banc Atlantis, N-O “le Suroit” Seamount 2, Atlantic Ocean, 34°22, 4′N, 30°27, 80′W, dredged from 1340 m, were acquired on loan from the Muséum National d’Histoire Naturelle, Paris (France). Specimens of *Cuvierina urceolaris* from Olango Island (dredged from 150 m), Balicasag Island (180 m) and Mactan Island (200 m), the Philippines, were purchased from Conchology Inc. In this way, material from the four families contained within the Cavolinioidea were examined.

### Scanning Electron Microscopy

Specimens of all species were fractured and ultrasonicated. Some samples were immersed in commercial bleach (4% active chlorine) for 1–4 min for further cleaning. Two specimens of *C. columnella* were immersed for longer times (15–20 min) in order to remove organic matter. Fragments of shells of *Cuvierina columnella*, *C. urceloaris*, *Cavolinia inflexa*, *Diacavolinia longirostris*, *Diacria trispinosa*, *D*. *quadridentata*, and *Clio pyramidata* were fixed for 3 days in 2.5% glutaraldehyde in sodium cacodylate buffer 0.1 M at pH = 7.4, and semi-demineralized in milli-Q water at pH = 5 for 2 days at room temperature. Specimens were coated with carbon (Hitachi UHS evaporator), prior to examination in either of the two FESEMs (Zeiss Leo Gemini 1530 and Zeiss Auriga Cross-Beam Station), belonging to the Centro de Instrumentación Científica (CIC) of the Universidad de Granada (UGR), Spain.

### Transmission Electron Microscopy

Cross section and plane-view samples for TEM analysis were prepared by cutting embedded shell pieces of *Cuvierina columnella* perpendicular and parallel to the shell surface, respectively. Thin cuts were first mechanically polished and subsequently thinned to electron transparency with a GATAN precision ion-polishing system (PIPS) at the Fritz-Haber Institute of the Max Planck Society in Berlin (Germany). TEM analysis was carried out using an image Cs corrected FEI Titan microscope that was operated at 300 kV.

Two TEM thin slices perpendicular to the internal shell surface, in parallel to the elongation of fibres, were cut out from a shell of *Cuvierina columnella* with the FIB-FESEM Zeiss Auriga Cross-Beam Station of the Centro de Investigación, Tecnología e Innovación (CITIUS) of the Universidad de Sevilla (US), Spain. They were later carbon-coated (Hitachi UHS evaporator) and viewed with TEM (Zeiss LEO 906E, Zeiss Libra 120 Plus and FEI Titan) at the CIC (UGR).

### Atomic Force Microscopy

Observations were performed in a Molecular Imaging Pico Plus microscope (CITIUS, US) in tapping mode, using an acoustic excitation module and a 10 μm × 10 μm scanner. All images were obtained from internal surfaces without any treatment other than gentle cleaning in distilled water. Topography, phase and amplitude signals were recorded and subsequently processed into images, height profiles or three-dimensional reconstructions using Gwyddion^36^.

## Additional Information

**How to cite this article**: Checa, A. G. *et al.* Biological strategy for the fabrication of highly ordered aragonite helices: the microstructure of the cavolinioidean gastropods. *Sci. Rep.* **6**, 25989; doi: 10.1038/srep25989 (2016).

## Supplementary Material

Supplementary Information
