# Supplementary material for: Biological strategy for the fabrication of highly ordered aragonite helices: the microstructure of the cavolinioidean gastropods
Source: Sci Rep. 2016 May 16;6:25989. doi: 10.1038/srep25989 (PMC4867615; doi:10.1038/srep25989)
Supplement: Supplementary Information [file srep25989-s1.pdf]

Biological strategy for the fabrication of highly ordered aragonite helices: the microstructure of the cavolinioidean gastropods

Antonio G. Checa[1,]*, Elena Macías-Sánchez[1], Joaquín Ramírez-Rico[2]

[1]Departamento de Estratigrafía y Paleontología, Universidad de Granada, Granada, 18071, Spain, and Instituto Andaluz de Ciencias de la Tierra (CSIC), Armilla 18100, Spain;

[2]Departamento de Física de la Materia Condensada, Universidad de Sevilla, 41012, Sevilla, Spain, and Instituto de Ciencia de Materiales de Sevilla (CSIC-Universidad de Sevilla), 41092 Sevilla, Spain.

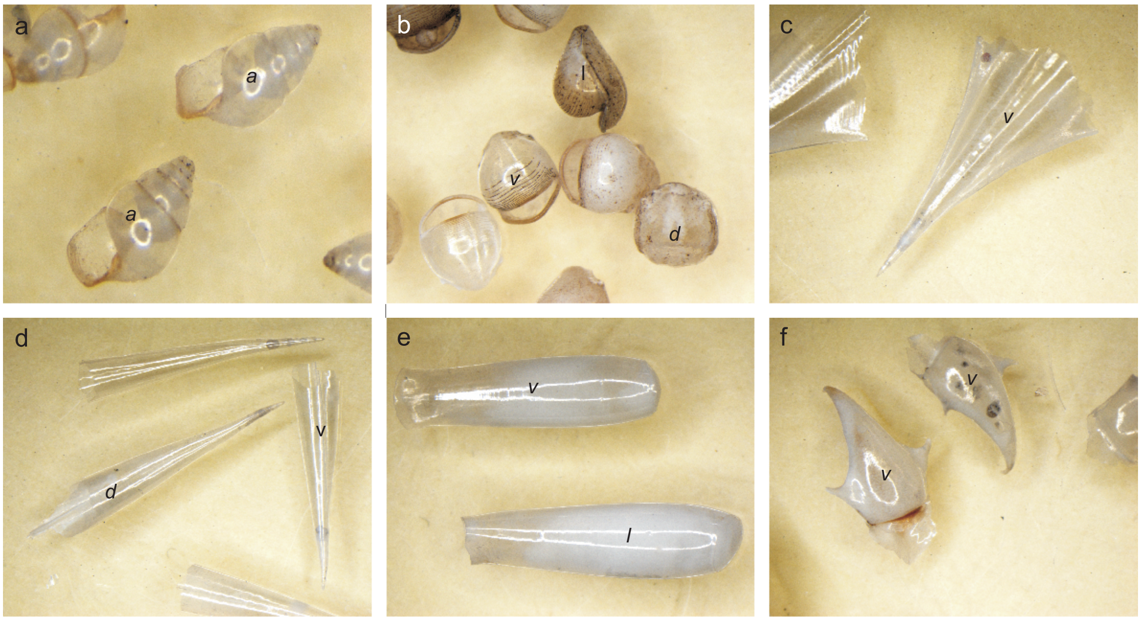

Figure S1. Shell morphologies of Euthecosomata gastropods. In contrast to the helically coiled Limacinoidea (a), the shells of the Cavolinoidea are straight (b-e) or curved (f). **a**. *Limacina bulimoides* (shell length, 1.1 mm). **b**. *Diacria quadridentata* (1.4-1.5 mm). **c**. *Clio pyramidata* (6.2 mm). **d**. *Creseis clava* (5.6-6.1 mm). **e**. *Cuvierina columnella* (8.8, 9.2 mm). **f**. *Cavolinia inflexa* (4.7, 5.0 mm). *a*, apertural view; *d*, dorsal view; *l*, lateral view; *v*, ventral view. All are optical micrographs.

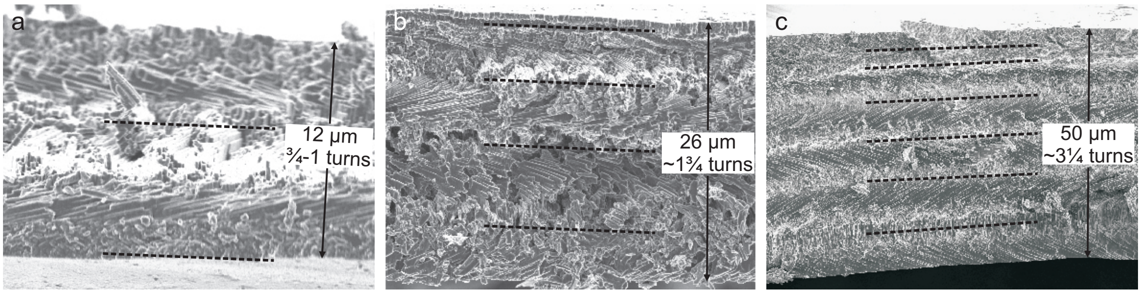

Figure S2. The shells of the Cavolinioidea display a direct, though not strict, relationship between the number of turns of the helical fibres and shell thickness. a. *Creseis clava*. **b**. *Clio pyramidata*. **c**. *Cuvierina columnella*. Broken lines are spaced at about half a whorl. Note the increasing spacing between them. SEM images.

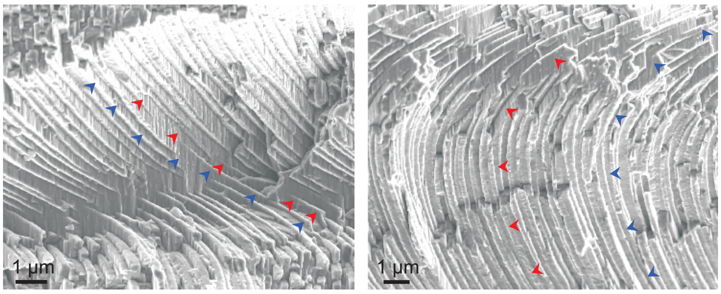

Figure S3. Continuity of the fibres in *Cuvierina columnella*. Two SEM details of fractures perpendicular to the external surfaces of the shells (to the top). The paths of particular fibres are marked with arrows in the same colour.

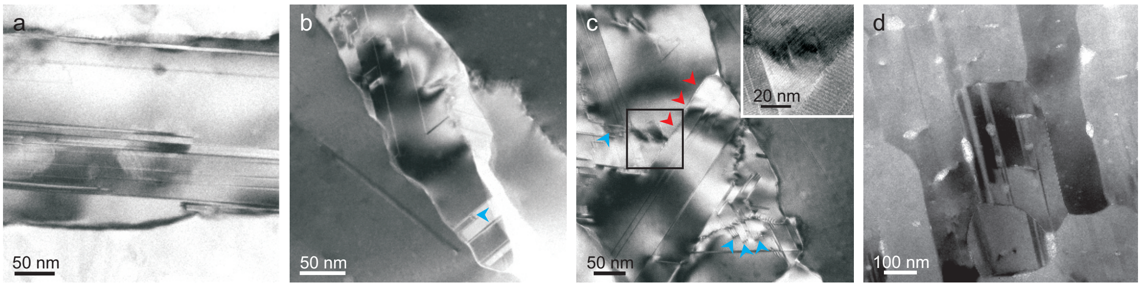

Figure S4. Distribution and types of twins in *Cuvierina columnella*, revealed by diffraction contrast under the TEM. a. Polysynthetic twins approximately parallel to the fibre axis. **b**, **c**. Combinations of polysynthetic and polycyclic twins. The arrangement in **c** is particularly complex, and there is a twin boundary that has become a crystal boundary (red arrows). The inset is a close-up of the framed area and shows the presence of nanometric polysynthetic twins. The blue arrows point to incoherent twin boundaries. **d**. Dense arrangement of twin planes; they are consistently oriented parallel to the coiling axis of the fibres. **a** to **c** are in-plane views and **d** is a cross-sectional view. Images **a** to **c** were acquired and kindly ceded by Dr. Marc Willinger (Fritz-Haber Institut, Berlin).

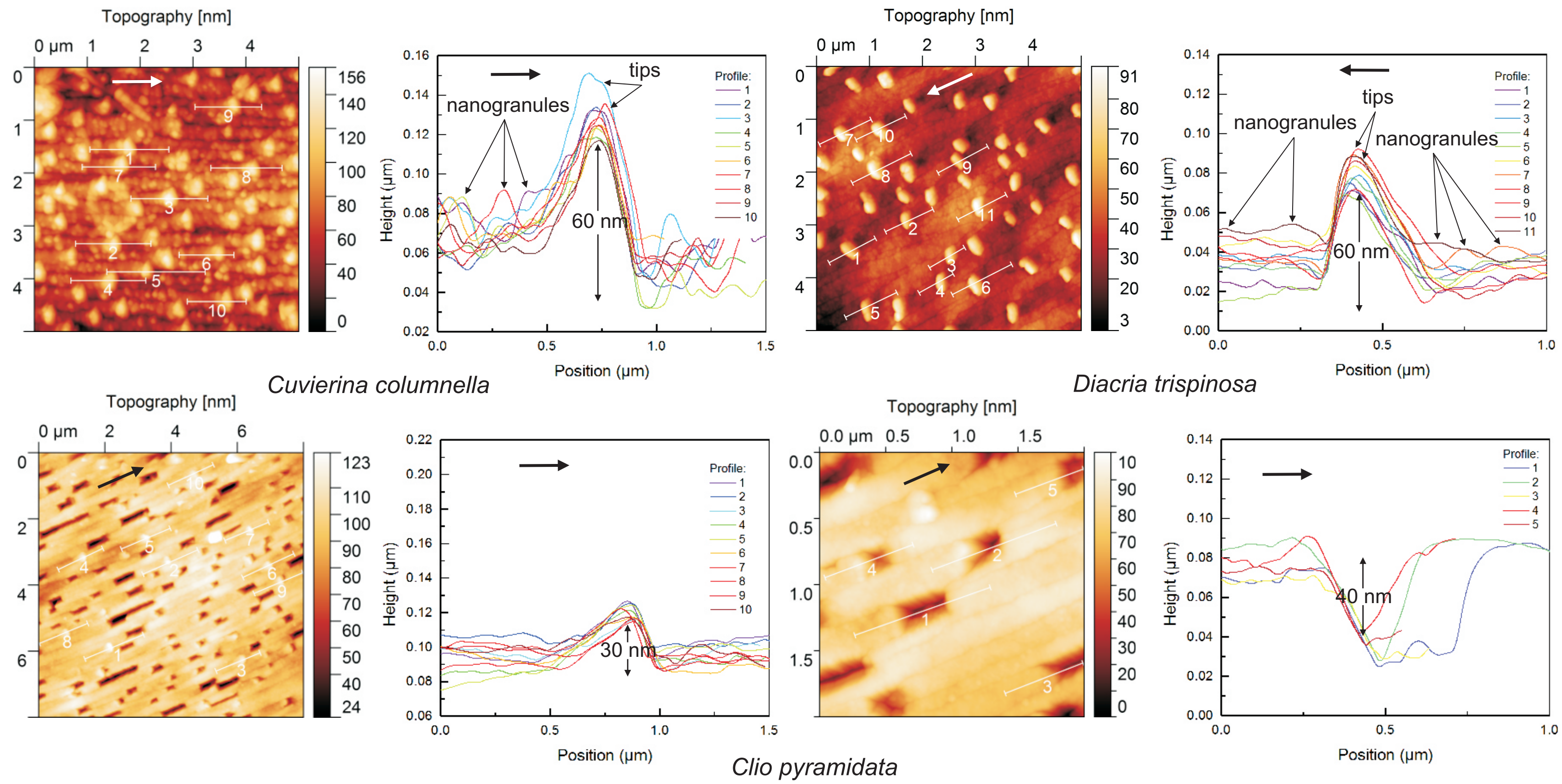

Figure S5. Topographic profiles on the internal shell surface along selected fibres of the species indicated, made with AFM. The posterior slope of the tips is consistently lower than the anterior slope. The small undulations are the profiles of nanoglobules (see Fig. 4) and the larger undulations correspond to the tips of fibres (indicated in the upper images). The lower right panels are sections along the depressions left by some tips which have detached from the shell. The profiles of the scars left on the fibres (left slopes) are flat surfaces with consistent slope values around 0.16-0.17, thus indicating that they may be crystallographic faces.

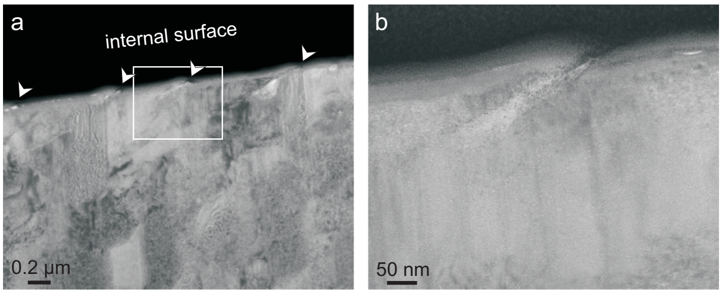

Figure S6. TEM section through the internal surface of the shell of *Cuvierina columnella*. a. The section has gone through the axes of fibres and cuts up to four tips (arrows). **b**. Close-up view of the area framed in **a**. The samples have been prepared with the FIB-SEM technique.

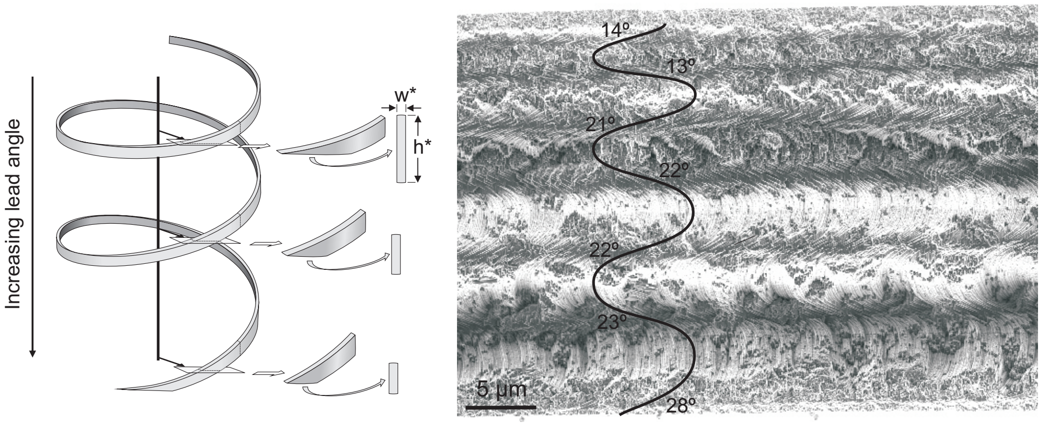

Figure S7. Influence of the lead angle on the relative dimensions of the sections of fibres when they are intersected by a plane perpendicular to the coiling axis (i.e. by the shell growth surface). When the lead angle increases, as observed in actual shells (right image), the width of the section with this plane (w*) remains constant, but the height (h*) decreases. Accordingly, towards more internal areas of the shell, more fibres can be accommodated within the same area of the growth surface. The right image is a SEM view of a fracture through the shell of *Cuvierina columnella* along the coiling axes of the helices, on which some lead angle values have been roughly estimated.

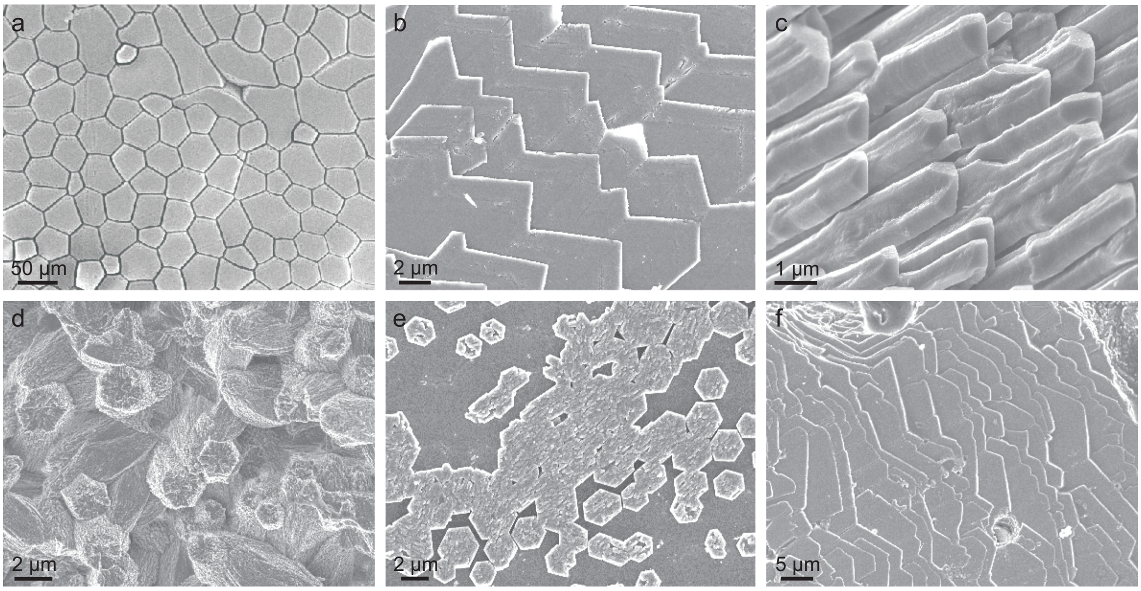

Figure S8. SEM views of the growth surfaces of selected molluscan microstructures. In all cases, the crystalline morphologies (boundaries and/or faces) are evident and individual crystals are homogeneous in composition. **a**. Calcitic prismatic microstructure of the bivalve *Pinna nobilis*. **b**. Calcitic foliated microstructure of the bivalve *Anomia ephippium*. **c**. Calcitic fibrous microstructure of the bivalve *Propeamussium dalli*. **d**. Granular prismatic microstructure of the cephalopod *Nautilus pompilius*. **e**. Nacre of the bivalve *Pteria hirundo*. **f**. Foliated aragonite of the monoplacophoran *Rokopella euglypta*.
